# Molecular basis of the interaction of the human tyrosine phosphatase PTPN3 with the hepatitis B virus core protein

**DOI:** 10.1038/s41598-020-79580-9

**Published:** 2021-01-13

**Authors:** Mariano Genera, Barbara Quioc-Salomon, Antonin Nourisson, Baptiste Colcombet-Cazenave, Ahmed Haouz, Ariel Mechaly, Mariette Matondo, Magalie Duchateau, Alexander König, Marc P. Windisch, Christine Neuveut, Nicolas Wolff, Célia Caillet-Saguy

**Affiliations:** 1 Channel-Receptors Unit, UMR 3571, CNRS, Institut Pasteur, 75015 Paris, France; 2 Complexité du Vivant, Sorbonne Université, 75005 Paris, France; 3 UMR 3569, CNRS, 75015 Paris, France; 4 Department of Virology, Institut Pasteur, Paris, France; 5 Université Paris Diderot, Sorbonne Paris Cité, Paris, France; 6 Crystallography Platform-C2RT, Department of Structural Biology and Chemistry, CNRS, UMR-3528, Institut Pasteur, 75015 Paris, France; 7 Proteomics Platform, Mass Spectrometry for Biology Utechs (MSBio), USR 2000, CNRS, Institut Pasteur, 75724 Paris, France; 8 Applied Molecular Virology Laboratory, Institut Pasteur Korea, 696 Sampyung-dong, Bundang-gu, Seongnam-si, Gyeonggi-do South Korea; 9 Present Address: Institute of Human Genetics, 141 rue de la Cardonille, 34090 Montpellier, France

**Keywords:** Proteins, Biophysical methods, High-throughput screening, Structure determination, Biochemistry, Biological techniques, Molecular biology

## Abstract

Interactions between the hepatitis B virus core protein (HBc) and host cell proteins are poorly understood, although they may be essential for the propagation of the virus and its pathogenicity. HBc has a C-terminal PDZ (PSD-95, Dlg1, ZO-1)-binding motif (PBM) that is responsible for interactions with host PDZ domain-containing proteins. In this work, we focused on the human protein tyrosine phosphatase non-receptor type 3 (PTPN3) and its interaction with HBc. We solved the crystal structure of the PDZ domain of PTPN3 in complex with the PBM of HBc, revealing a network of interactions specific to class I PDZ domains despite the presence of a C-terminal cysteine in this atypical PBM. We further showed that PTPN3 binds the HBc protein within capsids or as a homodimer. We demonstrate that overexpression of PTPN3 significantly affects HBV infection in HepG2 NTCP cells. Finally, we performed proteomics studies on both sides by pull-down assays and screening of a human PDZ domain library. We identified a pool of human PBM-containing proteins that might interact with PTPN3 in cells and that could be in competition with the HBc PBM during infection, and we also identified potential cellular partners of HBc through PDZ-PBM interactions. This study opens up many avenues of future investigations into the pathophysiology of HBV.

## Introduction

Hepatitis B virus (HBV) remains a major global health problem, with 257 million chronic HBV carriers and almost 1 million deaths estimated in 2015 (WHO). Despite available prophylactic vaccines, chronic hepatitis B is still incurable; existing treatments focus on stopping the spread of the virus thereby preventing disease progression. The acute infection can be asymptomatic or symptomatic, in some cases, resulting in fulminant hepatitis. Chronic infection can lead to the development of cirrhosis and hepatocellular carcinoma (HCC).

HBV is a small, enveloped DNA virus. The infectious particle contains a 3.2 kb partially double-stranded relaxed circular DNA genome (rcDNA) surrounded by the capsid composed by the core protein (HBc). The different steps of the viral life cycle are: the virus entry in the cell, the translocation of the nucleocapsid to the nuclear pore allowing the release of the rcDNA into the nucleoplasm and its ‘repair’ into covalently closed circular DNA (cccDNA), the transcription of the viral RNAs from the cccDNA, the export of the pgRNA to the cytoplasm and viral protein translation, the encapsidation of the pgRNA and its reverse transcription into rcDNA inside the capsids. Nucleocapsids are then enveloped at the endoplasmic reticulum and released, or alternatively, they return to the nucleus to form additional cccDNA molecules. The HBV capsid protein (HBc) has functions that surpass its structural role. Indeed, HBc has been implicated in most stages of the viral life cycle, including subcellular trafficking and release of the viral genome, pgRNA encapsidation, and reverse transcription^1^. Although HBV is relatively well studied, little is known about its HBc-mediated protein interactions with host proteins. To clarify the role of the virus structural protein in the pathogenesis of HBV-infected hepatocytes and to identify human proteins involved in important stages of the virus life cycle, the interactions with host-cell proteins have to be investigated.

HBc protein (183 residues) presents an N-terminal capsid assembly domain (residues 1–149)^2^ and an arginine-rich RNA-binding C-terminal domain (CTD, residues 150–183), which is dispensable for capsid assembly in vitro but is required for viral genome replication in cells^2^. The CTD also contains nuclear localization signals and cytoplasmic retention signals that regulate nuclear import and export of the HBV capsid^3^, and multiple serine and threonine residues that are susceptible of being phosphorylated^4^. Finally, the C-terminal residues of the CTD form a non-canonical class I PDZ-binding motif (PBM) with a C-terminal cysteine (–ESQC_COOH_; class I PBMs are defined as –[X–S/T–X–Φ], where Φ is any hydrophobic amino acid and X is any amino acid). This PBM has been shown to interact with the PDZ (PSD-95, Dlg1, ZO-1) domain of protein tyrosine phosphatase non-receptor type 3 (PTPN3)^5^ and GIPC1^6^. The level of HBV RNAs in HuH-7 cells overexpressing PTPN3 is reduced independently of the PBM-mediated interaction with HBc after transfection of an expression plasmid containing the HBV genome^5^. Similarly, PTPN3 is targeted through a PBM-mediated interaction by the oncogenic high-risk human papillomaviruses (HPVs)^7^. In this case, the deletion of the PBM of E6 in the HPV type 31 genome resulted in transfected cells that were significantly reduced in their growth rates and reduced in their viral copy numbers compared to keratinocytes transfected with wild-type genomes^8^. Since the deletion of the PBM of E6 eliminates the interaction with all of its PDZ targets, the full significance of any particular PDZ-containing target in the viral life cycle or the development of cancer remains open for investigation. It is possible that E6 degradation of PTPN3 contributes to such a phenotype, as shRNA to PTPN3 also produced a similar but less pronounced phenotype than E6. Thus, we propose that targeting PTPN3 through a PBM-PDZ interaction may be significant to the viral life cycle and/or in the oncogenic character of HBV. Notably, PTPN3 has been implicated in many cancers^9–14^, and it has been suggested that PTPN3 mutations and HBV may exert synergistic effects in the origin of the intrahepatic cholangiocarcinoma^15^. We further investigated the role of PTPN3 in a cell model system of Human hepatoma HepG2 NTCP cells stably expressing full-length PTPN3 under HBV infection to explore and to document the presence of this PBM in HBV core protein.

PTPN3 possesses three domains: an N-terminal FERM (band 4.1, ezrin, radixin, moesin) domain involved in localization to the cytoskeleton–membrane interface or interaction with transmembrane proteins, a central PDZ domain that mediates interaction with other proteins, and a C-terminal protein tyrosine phosphatase (PTP) domain bearing the catalytic activity. PTPN3 is a signalling protein, and its FERM and PDZ domains account for specificity in its functions. The FERM domain targets the protein to the plasma membrane providing spatial regulation, while the PDZ domain mediates interactions with specific partners and substrates, or anchors the phosphatase in multi-protein signalling complexes. A few ligands and substrates and known functions of PTPN3 have been reported^9,16–24^. However, its precise role in cell signalling has not yet been clearly established.

In the present study, we investigated the molecular mechanism of interaction between PTPN3 and HBc. To gain structural insights into the recognition of HBc by PTPN3, we solved the X-ray structure of the PDZ domain of PTPN3 (PTPN3-PDZ) in complex with the PBM of HBc (PBM-HBc), revealing a network of interactions specific to class I PDZ domains despite the atypical PBM of HBc containing a cysteine at its C-terminus. We confirmed the interaction by NMR. We showed that PTPN3 binds full-length HBc both as a homodimer and as a capsid-like particle, supporting a model in which the CTD comprising the PBM is at least transiently exposed at the surface of the capsids. To further explore the role of PTPN3 in the HBV life cycle, we report the effects of PTPN3 overexpression on HBV infected HepG2 NTCP cells. We also performed proteomic studies in two directions. Indeed, we performed pull-down assays with PTPN3-PDZ to detect endogenous PBM-containing proteins that potentially interact with PTPN3 in cells, and that could be displaced by the PBM-HBc during viral infection. We discuss several of the identified putative PTPN3 partners that have been previously reported as implicated in HBV infection and/or in HCC to propose potential signalling pathways disrupted by HBV and/or involved in HCC. We also performed a high-throughput analysis of the HBc-interacting partners through PDZ-PBM interactions, and we showed that HBV targets the same PDZ-containing proteins as the high-risk HPV oncovirus. Interestingly, PTPN3 has also been shown to be targeted by the high-risk human papillomavirus types 16 and 18 (HPV 16 and 18) through the PBM of the viral E6 oncoprotein^7^. We previously reported the similar binding affinities of the PBMs from E6 and HBc for PTPN3-PDZ (K_D_ of a few tens of micromolar) and the structure of PTPN3-PDZ in complex with the PBM of the HPV16 E6 protein (PBM-16E6)^25^. HBV and HPV 16 and 18 are both oncoviruses, and accumulating evidence suggests that PTPN3 plays a critical role in the progression of various human cancers (breast, lung, colorectal cancer, intrahepatic cholangiocarcinoma, hepatocellular carcinoma)^9–14^.

## Results

### The atypical PDZ-binding motif of HBc interacts with PTPN3-PDZ through a network specific to class I PDZ domains

PDZ domains can be grouped into three main specificity classes: class I recognizes a serine or threonine at position − 2 (P_−2_) within a C-terminal peptide sequence-motif defined as –[X–S/T–X–Φ_COOH_], where Φ is a hydrophobic residue and X is any residue; class II recognizes any hydrophobic residue at P_−2_ within a motif defined as –[X–Φ–X–Φ_COOH_]^26,27^; and class III, which recognizes the motif –[X–D/E–X–Φ_COOH_]^28^. The PBM-HBc is an atypical PBM because it features a cysteine at its C-terminus. All the sequence consensus classes commonly used to describe PBMs present a hydrophobic residue in the last C-terminal position. Although cysteine is often considered to be hydrophobic, its classification is ambiguous, as some consider it to be polar since there is a slight difference in electronegativity between S and H. However, as we previously reported, its affinity for PTPN3-PDZ is similar to the affinity of canonical class I PBMs such as the PBM-16E6, which presents a leucine at the C-terminus (HPV16 E6 C-terminal sequence ETQL)^25^.

**Figure 1:**
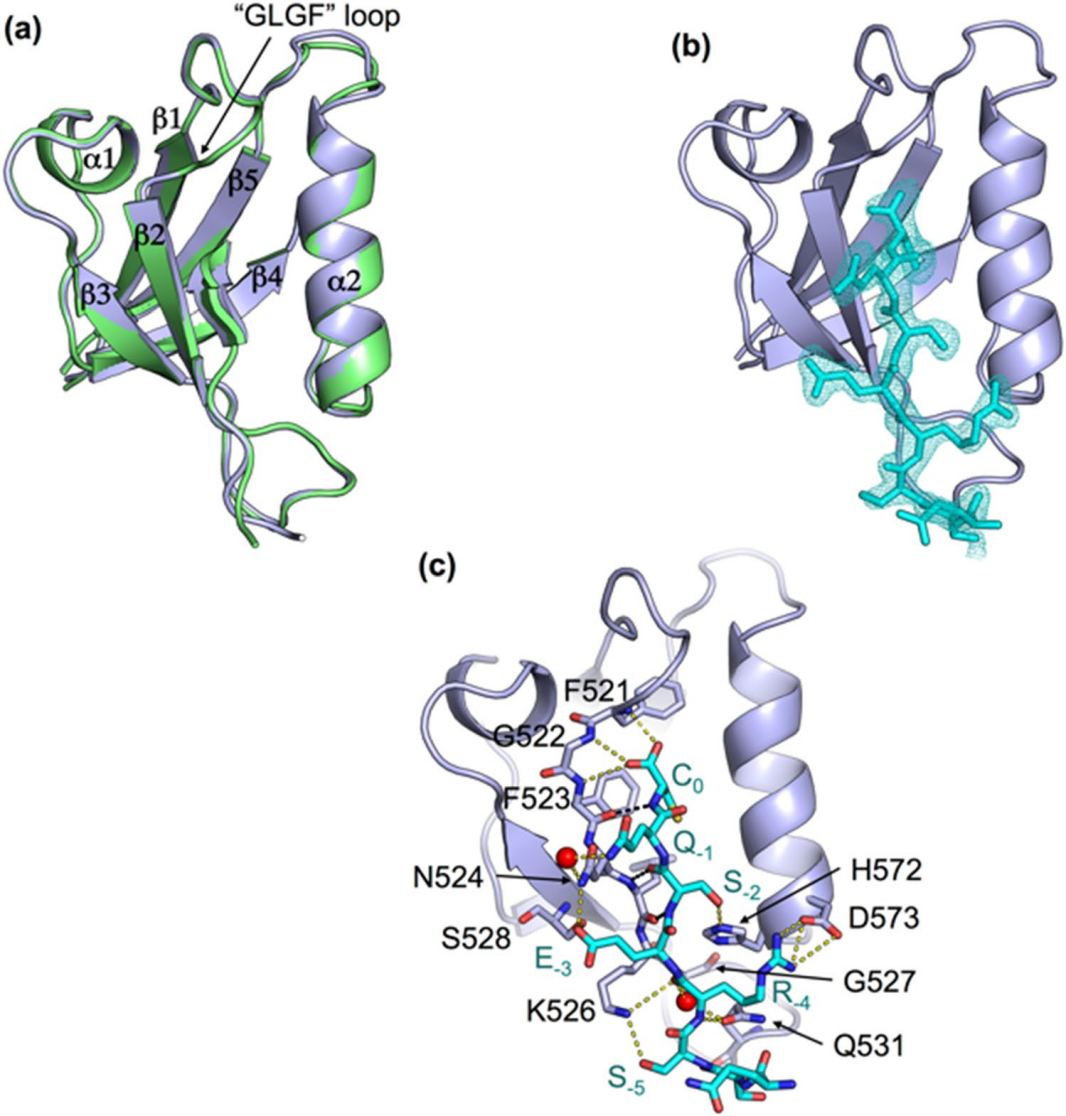
X-ray structure of PTPN3-PDZ bound to PBM-HBc. (**a**) Superimposition of the structures of PTPN3-PDZ bound to PBM-HBc (PDB ID: 6T36, light grey) and PBM-HPV16E6 (PDB ID: 6HKS, light green). (**b**) Representation of the density of the peptide in the binding groove of the PDZ domain. (**c**) Detailed views of the PTPN3-PDZ bound to PBM-HBc. Important residues are shown as sticks in CPK colors, molecules of water are represented as red spheres, and intermolecular H-bonds and polar contacts are shown as yellow dashed lines. Black dashed lines show the H-bond pairing with the β2-strand.

To decipher the molecular basis of recognition of the C-terminal sequence of HBc by PTPN3, we solved the crystal structure of the complex formed by PTPN3-PDZ and the PBM-HBc peptide (_NH2_RRRRSQSRESQC_COOH_) by molecular replacement at 1.86 Å resolution. The structure of the complex between PTPN3-PDZ and the class I PBM-16E6 (PDB ID: 6HKS) that we previously solved was used as a template (Fig. 1, Table 1). The structure factors and coordinates have been deposited in the Protein Data Bank under accession code 6T36. We compared the set of intermolecular bonds in both complexes to obtain structural insights on the binding mode of the unconventional HBc PBM.

**Table 1:** Data collection and refinement statistics.

	PTPN3-PDZ/ PBM-HBc
**Data collection**	
Space group	P 31 2 1
Unit cell (a, b, c) (Å)	75.58, 75.58, 46.59
α, β, γ (°)	90.00, 90.00, 120.00
Resolution (Å)	46.59–1.86 (1.96–1.86)
No. of reflections (total/unique)	67,510/13,198
Redundancy	5.1 (5.0)
Completeness (%)	99.4 (97.8)
*I*/σ (*I)*	17.38 (2.70)
*R-*meas^¶^ (%)	5.6 (57.5)
CC(1/2)	99.9 (97.7)
**Refinement**	
Resolution (Å)	29.35–1.86
No. reflections	13,066
*R*_work_†/*R*_free_‡	0.197/0.236
No. of protein atoms/ligand atoms	749/63
No. of solvent/hetero-atoms	68/1
Rmsd bond lengths (Å)	0.007
Rmsd bond angles (°)	0.836
*Wilson B*-factors	28.6
Ramachandran plot (favored/disallowed)*	96.9/0.0
PDB code	6T36

Values in parenthesis correspond to the highest resolution shell.

^¶^Rmeas = Σh(n/n−1)^1/2^Σi |Ii(*h*)—<I(h)>|/ΣhΣi Ii(*h*), where Ii(*h*) and <I(*h*)> are the ith and the mean measurement, respectively, of the intensity of reflection h.

^†^Rwork = Σh||Fobs(h)|−|Fcalc(h)||/Σh|Fobs(h)|, where Fobs(h) and Fcalc(h) are the observed and calculated structure factors, respectively. No I/σ cut-off was applied.

^‡^Rfree is the R-value obtained for a test set of reflections consisting of a randomly selected 5% subset of the data set excluded from refinement.

*Categories were defined by MolProbity.

As expected, the overall structures of PTPN3-PDZ in complex with PBM-HBc and PBM-16E6 are similar with a very low root mean square deviation (rmsd) of 0.18 Å for all the backbone atoms (Fig. 1a). PTPN3-PDZ adopts the typical PDZ fold comprising five β-strands and two α-helices. A clear electron density map was seen for the last eight C-terminal residues of PBM-HBc (underlined in the peptide sequence –RRRRSQSRESQC_COOH_). The last five residues are inserted into the binding groove (Fig. 1b). PBM-HBc binds PTPN3-PDZ in a conventional way, inserting into a hydrophobic cleft formed by the β2-strand, the α2-helix, and the “GLGF” loop (Fig. 1a) and pairing as an anti-parallel extension of the β2-strand, as observed for the PBM-16E6. Indeed, the backbone amide proton of the cysteine (C_0_) and the serine (S_−2_) and the backbone carbonyl oxygen of S_−2_ of the PBM-HBc are H-bonded to the β2 strand of PTPN3-PDZ through the backbone carbonyl oxygen of F523, L525, and the backbone amide proton of L525 respectively (black dashed lines in Fig. 1c).

**Figure 2:**
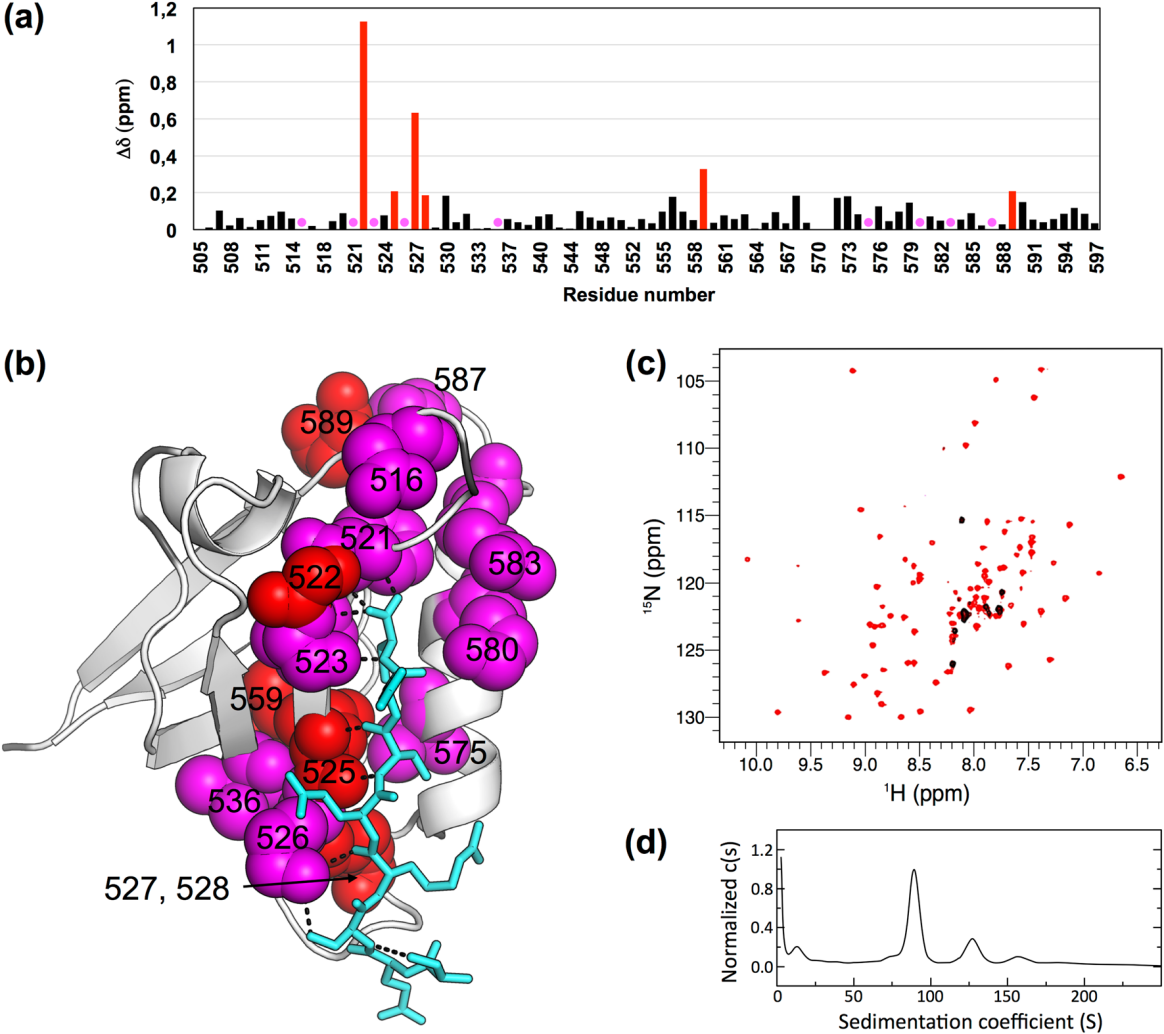
Δδ between free PTPN3-PDZ and PTPN3-PDZ bound to PBM-HBc. (**a**) Δδ values (^1^H, ^15^N) computed as ∆δ = [(∆δH)^2^ + (∆δN × 0.15)^2^]^1/2^. Red bars mark residues perturbed with ∆δ > 0.19 ppm (σ = 0.14 ppm); magenta circles mark residues of PTPN3-PDZ severely affected when binding to the PBM with signals that disappear from their original well-resolved position in the spectrum upon complex formation (**b**) Views of the structure of PTPN3-PDZ bound to PBM-HBc (PDB ID: 6T36) with the color depending on Δδ values. Red: ∆δ > 0.19 ppm; magenta: resonances absent in the complexed PTPN3-PDZ spectrum; grey: residues whose behaviour could not be safely defined, mainly because they fall in crowded spectral regions. Residues affected are represented as spheres. PBM-HBc peptide is represented as blue sticks with its H-bonds as dashed black lines. (**c**) HSQC NMR spectra of ^15^N-labelled PTPN3-PDZ free (in red) or in presence of an excess of full-length HBc (in black). (**d**) AUC diagram of the full-length HBc sample used for NMR experiments.

PTPN3-PDZ possesses the interaction network specific to class I PDZ domains, which differ from other classes by the nature of the residue at P_−2_. The key interactions of the PBM residues at positions 0 and − 2 with PTPN3-PDZ are identical to those we reported for the complex formed between PTPN3-PDZ and the PBM-16E6, in agreement with the same range of affinity of PTPN3-PDZ for PBM-HBc and classical PBMs^25^. The carboxylate of the C-terminal cysteine (C_0_) of PBM-HBc forms three H bonds with the amide protons of F521, G522, and F523 of the ‘GLGF’ motif on PTPN3-PDZ (Fig. 1c). The side-chain of the C_0_ occupies the same position as the leucine side-chain in the complex with PBM-16E6 (Figs. 1b, 2c). The space between the isoleucine 579 side-chain and the C_0_ permits to accommodate the cysteine side-chain in this hydrophobic environment without additional polar contacts to maintain its position. In position − 2, the hydroxyl group of the serine (S_−2_) forms a H-bond with the Nε2 of the conserved H572 from the α2 helix, as those we observed for T_−2_ in the PTPN3-PDZ/PBM-16E6 complex.

Interestingly, the same residues are found in positions − 1, − 3, and − 4 (Q, E, and R, respectively) in both viral PBMs, PBM-16E6, and PBM-HBc. Accordingly, we observed the same network of polar contacts in both complexes (Fig. 1c). In position − 1, the εNH2 of the exposed glutamine (Q_−1_) side-chain is H-bonded to the side-chain carbonyl and the terminal amine of N524 of the β2-strand through a water molecule. The hydroxyl of S538 and the amine proton of N524 form H-bonds with the side-chain carboxylate of the glutamate in position − 3 (E_−3_), and the carboxylate oxygens of D573 at the beginning of the α2 helix form ionic bonds with the guanidinium nitrogens of arginine at position − 4 (R_−4_) (Fig. 1c). These bonds involving residues at positions − 3 and − 4 are known to modulate the affinity of the PDZ domain for PBM sequence while they do not strictly form part of the PBM^25,29–31^. A few water molecules conserved in the two complexes participate as well in the PBM binding network, such as the one making a bridge between R_−4_ and G527 and Q531 of the β2–β3 loop. Moreover, the backbone carboxyl oxygen of R_−4_ forms H-bonds with the side-chain amine of K526, which is, in turn, H-bonded to the hydroxyl of the serine of the PBM-HBc at position − 5 (S_−5_) (Fig. 1c). Finally, the backbone amide of R_−4_ forms a H-bond with the side-chain carboxyl of Q531, whose amine group is also bonded to the backbone carboxyl of S_−8_. In contrast, a water molecule makes the bridge with the threonine at position − 7 (T_−7_) within the complex with HPV16E6 PBM.

Thus, we determined that a C-terminal cysteine does not affect the PBM binding, considering the similar binding affinities of the PBM-HBc and the PBM-16E6 for PTPN3-PDZ, and their similar bonding patterns, typical of class I PDZ complexes.

### The PDZ-binding motif of HBc induces direct perturbations on residues at the PBM binding site of PTPN3-PDZ and in its vicinity

To provide insights at the atomic level on the residues of PTPN3 affected upon interaction with HBc, and on potential distal effects, we probed the binding of PTPN3-PDZ to the PBM-HBc by solution NMR experiments. ^1^H, ^15^N heteronuclear single quantum coherence (HSQC) spectra were recorded on ^15^N-labeled PTPN3-PDZ in its free form and in the presence of the unlabeled PBM-HBc peptide. We assigned the signals using the previous assignment of the backbone of the PTPN3-PDZ complexed with PBM-16E6 (BMRB entry 27645), and we analyzed the chemical shift perturbations (Δδ) of the ^1^H, ^15^N signals of PTPN3-PDZ induced by the binding to PBM-HBc. Two types of signals were used for the analysis (Fig. 2a): (1) signals experiencing significant chemical shift changes (Δδ > 0.19 ppm; standard deviation σ = 0.14 ppm); (2) signals experiencing large chemical shift changes or severe line broadening effects caused by exchange, which disappear upon complex formation. Six residues belonging to signals of type 1, G522, L525, G527, G528, I559, E589, are shown in red in Fig. 2a,b, while 9 residues of signals of type 2, D516, F521, F523, K526, V536, V575, K580, R583, S587, are colored in magenta in Fig. 2a,b. Seven residues, D505, D518, I550, K552, H570, T571, R588, whose behaviour could not be safely defined mainly because they fall in crowded spectral regions, are colored in gray (Fig. 2b).

As expected, signals of the residues located in the PBM binding site such as L525, K526, and G527 of the β2-strand; K580, R583 and V575 of the helix α2; or F521, G522, F523 of the “GLGF motif” are among the most affected when the complex with PBM-HBc is formed (Fig. 2b). G528 in the β2–β3 loop, V536 on the β3 strand, and the S587 and E589 in the α2–β5 loop are in close proximity to the PBM binding site and are also affected. In addition, L525, in direct contact with the ligand, is located 3.9 Å away from I559 on the β4 strand, likely transmitting the perturbation through hydrophobic contacts between the two. D516 also experiences a strong effect upon PBM binding due to a network of water molecules that links it to the carboxylate of C_0_. Thus, direct and distal perturbations are induced upon PBM binding, as we previously observed in PTPN3 and PTPN4 PDZ domains^25,32^.

### PTPN3-PDZ binds to full-length HBc assembled into CLPs

We then tested the complex formation between PTPN3-PDZ and the full-length HBc (Fig. 2c). ^1^H, ^15^N HSQC-TROSY spectra were recorded on ^15^N-labeled PTPN3-PDZ in its free form (red signals in Fig. 2c) and in the presence of the unlabeled HBc full-length protein at different ratios. After stepwise additions of HBc to a final ratio 1:19.4 (black signals in Fig. 2c), nearly all of the TROSY resonances observed with free PTPN3-PDZ had disappeared, almost certainly because transverse relaxation in the complexes was too fast to observe NMR signals even with the TROSY effect. Indeed, wild type (WT) HBc proteins form homodimers and self-assemble into capsids^33^, and the full-length HBc expressed in *Escherichia coli* mainly assembles into capsid-like particles (CLPs) of 3 to 5 million Da, as measured by analytical ultracentrifugation (AUC) (Fig. 2d). Indeed, we observed by AUC a large peak corresponding to the main species in our sample with a sedimentation coefficient of 85 S, in agreement with the values previously reported for HBV CLPs at 82.5 S^34^. The molecular tumbling of PTPN3-PDZ is drastically slowed down when forming high molecular weight complexes with the CLPs, which induced the broadening of the linewidths of the PTPN3-PDZ signals beyond detection. Yet, the TROSY spectra of the complexes did contain a few peaks, which we attributed to regions of PTPN3-PDZ that remained mobile, probably the N-terminal and C-terminal extremities. These results indicate that PTPN3-PDZ can bind the CTD of full-length HBc within CLPs in vitro.

### PTPN3 binds to the HBc protein within capsids as well as to the assembly-deficient mutant Y132A

**Figure 3:**
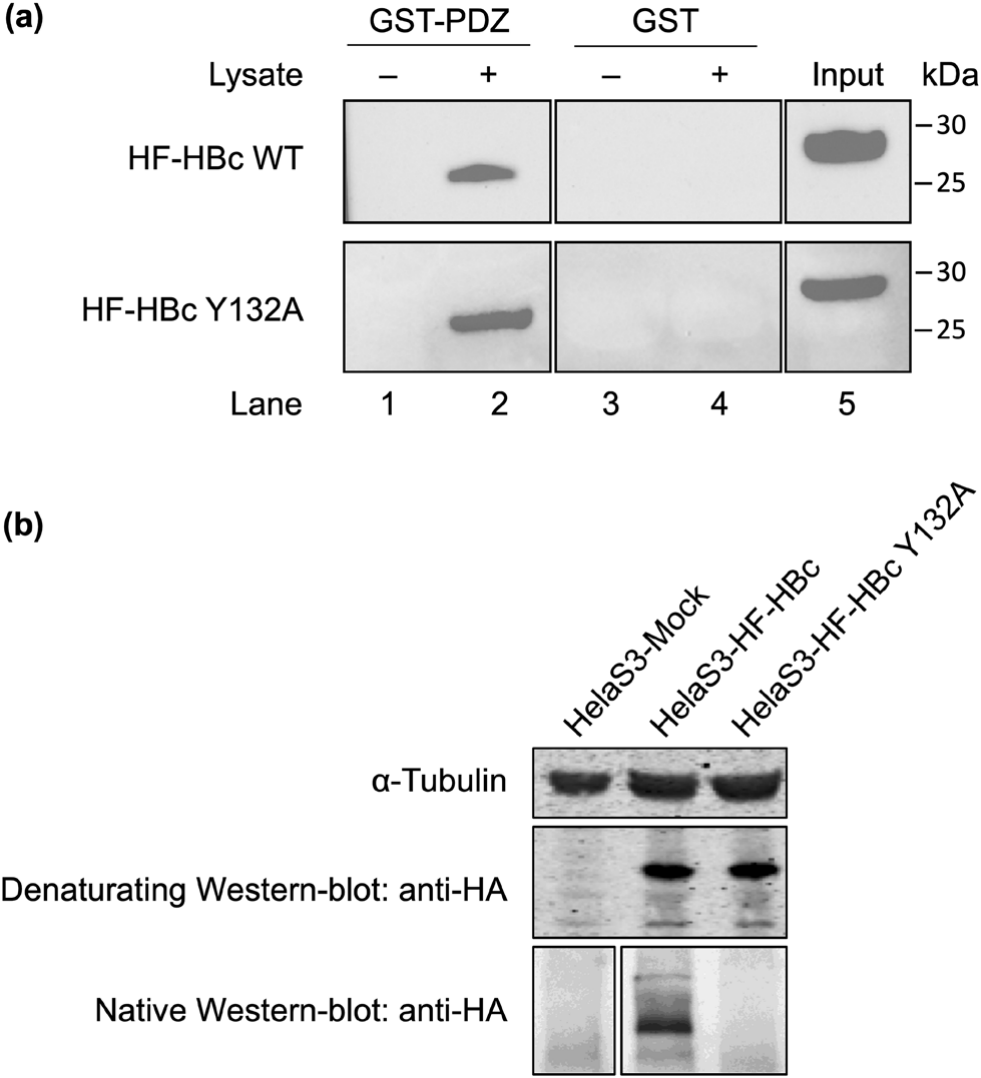
GST pull-down assay of GST-tagged PTPN3 PDZ domain against HeLa S3 cell lysate overexpressing HBc. (**a**) GST (negative control) and GST fusion proteins were bound to the same amount of glutathione-agarose beads and allowed to interact with HF-tagged HBc protein from lysates of HeLa S3 cells overexpressing either the HF-tagged full-length WT HBc or the Y132A mutant. Samples were washed and analyzed by Western blot with anti-HA antibodies. (upper part) Lanes 1 and 2: GST-PTPN3-PDZ in absence or presence of lysate containing HF-HBc WT, respectively; Lanes 3 and 4: GST in absence or presence of lysate containing HF-HBc WT respectively; Lane 5 shows the signal of HF-HBc WT in lysates of HeLa S3 cell lysate*.* (lower part) Lanes 1 and 2: GST-PTPN3-PDZ in absence or presence of lysate containing HF-HBc Y132A, respectively; Lanes 3 and 4: GST in absence or presence of lysate containing HF-HBc Y132A respectively; Lane 5 shows the signal of HF–HBc Y132A in lysates of HeLa S3 cell lysate. (right) Ticks and labels indicate molecular weight markers. (**b**) Native Western blot on the HeLa S3 HF-HBc WT and HF-HBc Y132A using the anti-HA antibody. Note that blots represent discontiguous panels from the gels when black line delimitation is present. The images have been cropped to frame the relevant region for presentation.

To investigate whether the PBM at the end of CTD of HBc is accessible to PTPN3 binding during capsid assembly, a glutathione *S*-transferase (GST) pull-down in vitro assay was performed with lysates of HeLa S3 cells overexpressing the HA-Flag-tagged either wild-type full-length HBc (HF-HBc WT) or its Y132A mutant (HF-HBc Y132A) that forms dimers but is unable to assemble into capsids^35^ (Fig. 3a; Supporting Information Fig. 1). GST protein was used as a negative control. The expression of HF-HBc WT and HF-HBc Y132A in the cell lysates was verified by Western blot using the anti-HA antibody (Fig. 3a, lane 5). We also confirmed, using native Western blot, the presence of capsid in the HeLaS3 HBc WT cell lysate and its absence in the HeLaS3 HBc Y132A cell lysate (Fig. 3b).

The GST alone and its fusion with PTPN3-PDZ (GST-PTPN3-PDZ), used as baits, were immobilized on glutathione beads and tested for their ability to pull down HF-HBc WT by Western blot using the anti-HA antibody. After washing, identical amounts of beads were analysed for the presence of the tagged proteins, and HBc WT was detected only in interaction with GST-PTPN3-PDZ (Fig. 3a upper part, lane 2). Thus, the interaction between HF-HBc and GST-PTPN3-PDZ is specific to PTPN3-PDZ.

Similarly, GST-PTPN3-PDZ, but not GST alone, was able to bind to HF-HBc Y132A as efficiently as HF-HBc WT (Fig. 3a lower part, lane 2). These results demonstrate that PTPN3-PDZ interacts directly with the HBc protein both in the context of the full-length protein within capsids and in the dimeric form, and suggest that the interaction with PTPN3-PDZ could occur at different steps of viral infection.

### PTPN3 overexpression has multiple effects on HBV infection in hepatoma cells

**Figure 4:**
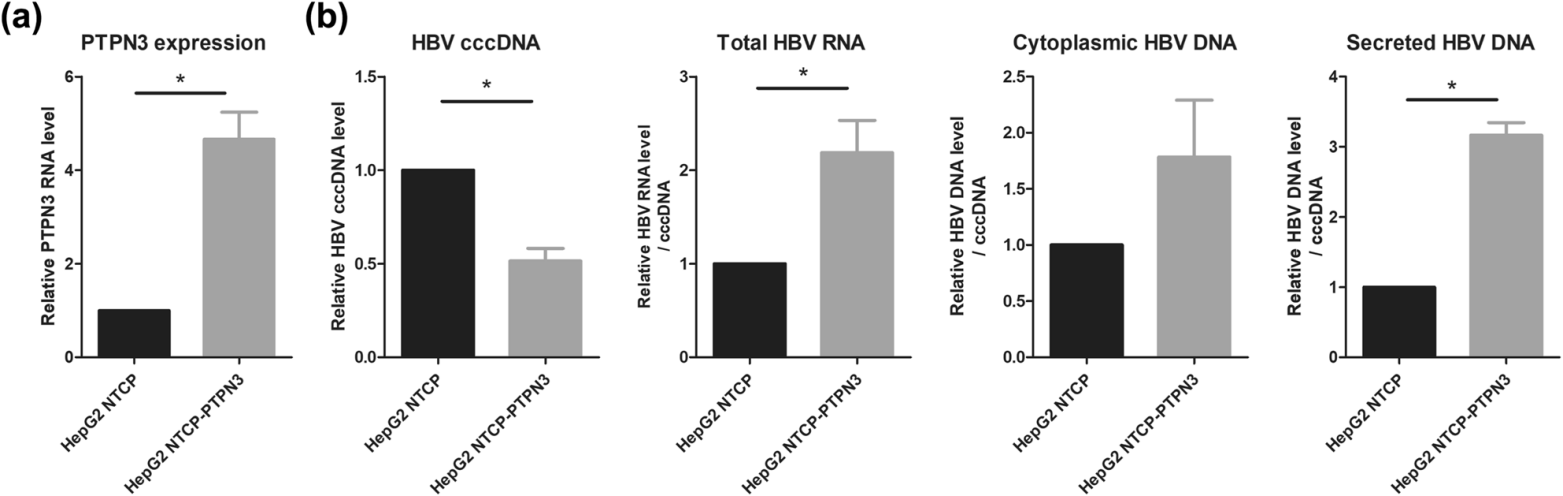
Effects of PTPN3 overexpression on HBV life cycle. (**a**) PTPN3 expression in HepG2 NTCP or HepG2 NTCP-PTPN3 cells was tested by RT-qPCR. (**b**) HepG2 NTCP or HepG2 NTCP-PTPN3 cells were infected at a multiplicity of infection (MOI) of 100 and 7 days after infection, effect of PTPN3 expression was analyzed: (left panel) cccDNA levels were analyzed by qPCR after nucleocytoplasmic fractioning; (second panel) HBV total RNA was analyzed and quantified by RT-qPCR; (third panel) cytoplasmic HBV DNA was quantified by qPCR after nucleocytoplasmic fractionation; (right panel) secreted HBV DNA was quantified by qPCR after preS1 immunoprecipitation. Results are expressed as the mean of six independent experiments, and error bars represent standard error of the mean (SEM). *p* values were determined by Wilcoxon matched paired test *p < 0.05.

Human hepatoma HepG2 NTCP cells stably expressing full-length PTPN3 (HepG2 NTCP-PTPN3) were established to monitor the effect of PTPN3 on HBV infection. Overexpression of PTPN3 was assessed by the quantification of PTPN3 RNA in HepG2 NTCP-PTPN3 compared to HepG2 NTCP and by Western blot analysis (Fig. 4a). Neither cell death nor morphological differences were observed in HepG2 NTCP-PTPN3 cells compared to HepG2 NTCP cells. This indicates that PTPN3 overexpression is well tolerated by HepG2 NTCP cells.

Because HBc may be involved in different steps of the HBV life cycle, from the first steps of infection to viral RNA transcription, encapsidation, and reverse transcription, we studied the impact of PTPN3 on HBV replication by following multiple virological parameters after HepG2 NTCP-PTPN3 and HepG2 NTCP infection. We measured the level of cccDNA using qPCR, HBV transcription using RT-qPCR, and calculated the ratio HBV RNA/cccDNA to evaluate cccDNA transcriptional activity. We also assessed HBV replication by measuring cytoplasmic RC-DNA by qPCR, and finally, we monitored HBV secretion by calculating the ratio secreted RC-DNA/cytoplasmic RC-DNA.

We observed a twofold decrease of cccDNA levels in HepG2 NTCP-PTPN3 compared to HepG2 NTCP (Fig. 4b). This might be due to a default of DNA import through the capsids on the nucleus or an effect of PTPN3 on capsid stability. However, a twofold increase in viral RNA and a threefold increase of secreted particles were in parallel also observed in PTPN3 overexpression conditions (Fig. 4b). These results indicate a significant role of PTPN3 in HBV replication.

### PTPN3 targets multiple PBM-containing binding partners in cells

We aimed at identifying new endogenous partner proteins of PTPN3 through a PDZ-PBM interaction to highlight potential biological pathways involving the phosphatase. We performed pull-down analyses using PTPN3-PDZ as bait and HeLa S3 cell lysate. Purified GST-PTPN3-PDZ and GST alone were incubated with the soluble fraction of HeLa S3 cell lysates. The interaction partners recovered in each sample were then identified by liquid chromatography (LC)-Mass Spectrometry (MS)/MS. In total, 348 and 54 different proteins, common between triplicates, were identified with GST-PTPN3-PDZ and GST alone, respectively. 326 proteins were specifically detected in GST-PTPN3-PDZ samples and were absent from GST controls (Supporting Information Table S1). These proteins are potential PTPN3 interactants through a PDZ/PBM interaction. Then, we narrowed our list of candidate proteins by selecting those that feature a PBM at their C-terminus. We shortlisted 104 potential PBM-containing proteins: 40 of class I (S/T–X–ϕ_COOH_), 62 of class II (Φ–X–Φ_COOH_), and 2 of class III (D/E–X–Φ_COOH_) with Φ: V, I, L, A, G, W, C, M, F (Supporting Information Table S2). PTPN3-PDZ is known to bind preferentially class I PBMs, so we focused on the 40 class I PBM-containing partners. Chronic infection with HBV can lead to the development of hepatocellular carcinoma (HCC)^36^. Therefore, we searched for potential links between the 40 class I PBM-containing partners and HBV and/or HCC. Only Vangl1 (planar cell polarity protein 1), a component of the Wnt/PCP pathway, was known to bind PTPN3, although it was not determined if the interaction occurred via its PBM^37^. Vangl1 is dysregulated in human cancers^38^. Interestingly, Vangl1 has been reported to be upregulated in the HBV-related cancer HCC, and associated with the invasion capacity of HCC cells^39^. Several other PTPN3-PDZ partners identified in our analysis have been linked to HCC. The nuclear transport receptor Exportin 2 is overexpressed in HCC, and its upregulation correlates with de-differentiation, proliferation, and poor prognosis^40^. The PTPN3-PDZ domain also binds Podocalyxin, whose expression is increased in HCC, and participates in migration and invasion processes^41^. Acot8 is often upregulated in HCC patients^42^, whereas its silencing reduces in vitro tumorigenesis in HCC cells.

Moreover, among the PTPN3 partners identified, some are directly linked to HBV infection. COX-2 is upregulated by HBV^43^, and its expression is also increased in the liver of cancer patients with chronic HBV infection. COX-2 might represent an important cellular effector of the HBV protein HBx, contributing to HBV-associated hepatocarcinogenesis^44^. Interestingly, hnRNP A0, another PTPN3 partner, is implicated in the LPS-induced post-transcriptional regulation of COX-2 mRNA and specifically bind COX-2 mRNA^45^. The PTPN3 binder 2′,3′-Cyclic Nucleotide 3′-Phosphodiesterase (CNP) is a gene stimulated by interferon. It also strongly inhibits HBV production by blocking viral protein synthesis and reducing viral RNAs, respectively^46^. In patients with chronic hepatitis B, CNP is expressed in most hepatocytes of HBV-infected liver specimens. Inactivation of CNP expression moderately enhances viral production in HepG2.2.15 cells treated with IFN-α. Thus, CNP could be an intermediary of the response induced by interferon against HBV. PTB, the polypyrimidine tract-binding protein, is another interesting PBM-containing partner of PTPN3. Indeed, PTB can bind to the HBV PRE (post-transcriptional regulatory element), an RNA element that facilitates the export of unspliced mRNAs of the nucleus. Thus, PTB could be important for the PRE activity and may function as an export factor for mRNAs containing PRE^47^.

In sum, we have identified potential PTPN3 PBM-containing cellular partners. Some of them have been reported in HCC and/or in HBV infection and could be displaced by HBc during HBV infection by competition with PBM-HBc. These results might provide some insight into the putative functions of viral proteins but should be interpreted with great care. Only studies in more biologically relevant systems will be more conclusive.

### HBV targets the same large pool of human PDZ-containing proteins as the oncogenic high-risk HPV

**Figure 5:**
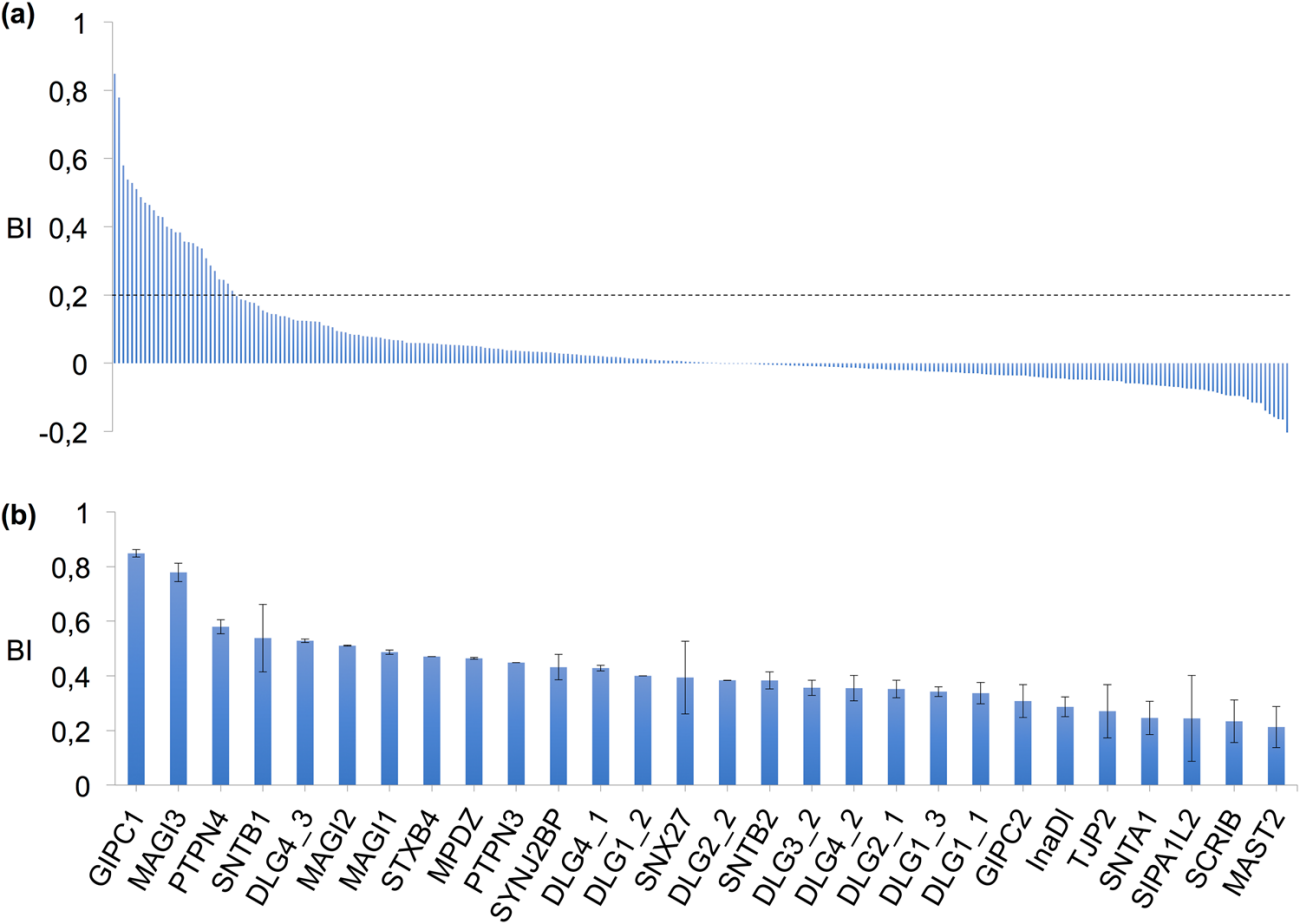
PDZome binding of HBc investigated by in vitro holdup protein-peptide binding assays. (**a**) PDZome-binding profile of PBM-HBc. PDZ domains are ranked on the basis of their BIs for the PBM-HBc peptide. A dashed line indicates the cut-off for a significant PDZ–PBM interaction (BI > 0.2). (**b**) Zoom-in on the 28 HBc binders with a BI > 0.2. The data and error bars are representative of two independent experiments.

To investigate the recognition specificity of the PBM-HBc against all human PDZ domains (the PDZome), we used the automated high-throughput holdup assay, which allows measuring binding intensities (BIs) for a large number of PDZ domain-PBM pairs^48^. We used an updated PDZome library that contains all the 266 known human PDZ domains^49^. The PDZome-binding profile (Fig. 5a) represents the individual BIs of each PDZ domain for the PBM-HBc peptide (peptide sequence with the PBM underlined: RRRRSQSRESQC). The binding intensities are directly linked to the PBM/PDZ affinities. Using BI = 0.2 as the minimum threshold for a significant PDZ-peptide interaction^25^, 28 potential HBc binders containing PDZ domains were identified (Fig. 5b, Table 2). This dataset represents about 10% of the human PDZome targeted by PBM-HBc with high-to-medium affinities. The best binders, GIPC1 and PTPN3, have already been reported as targets of the HBV core protein through PDZ/PBM interactions^5,6^. PBM-HBc also bound the PDZ domain of the non-receptor protein tyrosine phosphatase 4 (PTPN4), the closely related isozyme of PTPN3, and the PDZ domain of GIPC2, closely related to GIPC1. The interacting proteins include various PDZ proteins targeted by other oncoviruses. The membrane-associated guanylate kinase, WW and PDZ domain-containing proteins 1, 2, and 3 (MAGI1, MAGI2, and MAGI3), the discs-large homolog 1 and 4 (DLG1 and DLG4), the protein scribble homolog (SCRIB), the multi-PDZ domain scaffolding protein (MPDZ), the INADL protein (InaDl) and the Sorting Nexin 27 (SNX27)^50^ are all targeted by HPV types 16 and 18 through their E6 proteins^51^. PTPN3 and GIPC1 have also been reported as E6 binding partners. Thus, the same large pool of human PDZ-containing proteins is targeted by the oncoviruses HBV and the high-risk HPVs. Additionally, DLG1, MAGI1 and 3, and SCRIB are also targeted by another oncovirus, the Human T Lymphotropic Virus Type I (HTLV-I), through the PBM of its Tax protein. Likewise, non-oncogenic viruses target some of the PDZ-containing proteins that bind the PBM-HBc. Indeed, Adenovirus binds DLG1, MAGI-1, MPDZ, and the tight junction protein ZO-2 (TJP2) through the PBM of the early 4 ORF1 protein^51^. Then, the Microtubule-associated serine/threonine-protein kinase 2 (MAST2), the discs-large homolog 2 (DLG2), and PTPN4 interact with the PBM of the protein G of Rabies virus, and the beta-1-syntrophin (SNTB1) binds the HTLV-I Tax PBM^52^. All these HBc binders identified through the holdup assay and already reported as targets of other viral PBMs are good candidates for validation in cells.

**Table 2:**
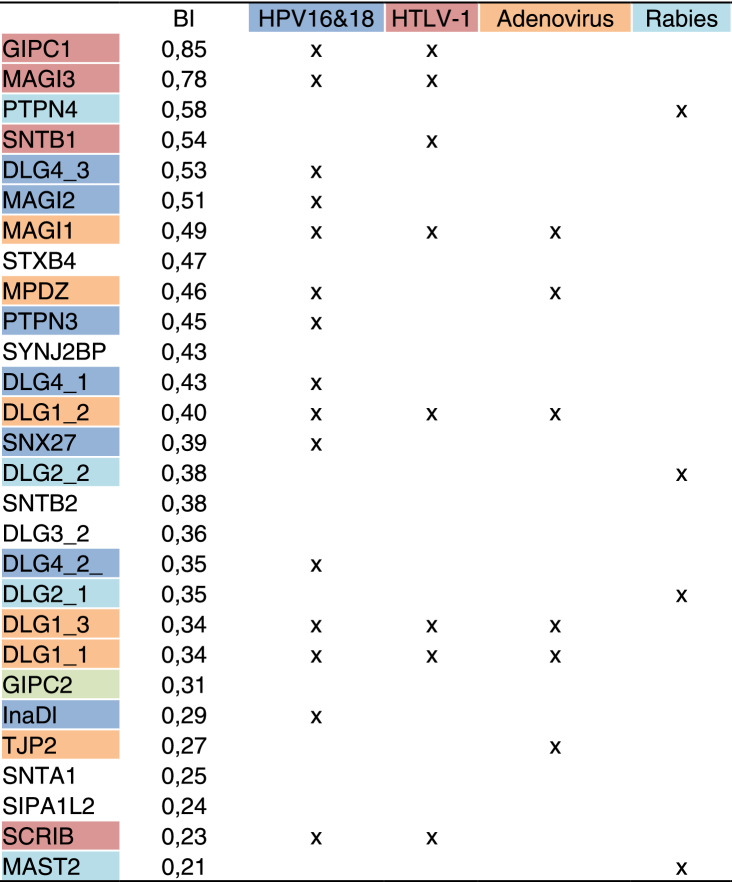
Best binders of HBc PBM in the holdup assay. Binding intensities (BIs) and occurrences between HBV and other viruses are reported.

The 6 remaining binders have not been reported to be targeted by viruses. These are the discs-large homolog 3 (DLG3), the alpha-1 and beta-2-syntrophin **(**SNTA1 and SNTB2), the Signal-induced proliferation-associated 1-like protein 2 (SIPA1L2), the Syntaxin-binding protein 4 (STXB4), and the Synaptojanin-2-binding protein (SYNJ2BP). They might be new relevant binders of PBM-HBc, or they interact because of PDZ domain homology, as in DLG3 with the DLG family and SNTA1 and SNTB2 with the syntrophin family. Alternatively, they might emerge from the degenerate specificity of PBMs or the promiscuity of PDZ/PBM interactions. Thus, the holdup assay enabled us to identify new host proteins potentially targeted by HBc during infection in addition to PTPN3 and GIPC1.

## Discussion

Thus far, only a few interactions of HBc with host proteins have been described. There are the ones mediated by the nuclear localization signals (NLS) present in the CTD of HBc, which can bind to importin αβ complexes and target the capsid to the nuclear pores^3^. Several host kinases have also been proposed as candidates for CTD phosphorylation^53^, and two human PDZ-containing proteins GIPC1 and PTPN3^5,6^ are known to bind the PBM of HBc. GIPC1 couples other proteins to myosin VI movements and participates in the recycling of membrane receptors^54^, and has been proposed to be involved in short intracellular transport of HBc proteins or capsids from the cytoplasm to the nucleus^6^. Its PDZ domain is targeted by at least two other viral proteins: Tax protein of HTLV-1^55^ and E6 protein of HPV18^56^. Concerning PTPN3, the precise role of the interaction between HBc and PTPN3 in HBV pathogenesis is still unknown.

In this study, we found that the effects of PTPN3 overexpression on HBV infection in hepatoma cells are multiple, suggesting that PTPN3 could have a significant and pleiotropic role in the HBV replication. In particular, we observed a decrease of cccDNA levels during HBV infection in HepG2 NTCP-PTPN3 compared to HepG2 NTCP. This might be due to a default of DNA import to the nucleus or an effect of PTPN3 on capsid stability or phosphorylation. We showed that PTPN3 binds the HBc CTD within CLPs and capsids in vitro. We hypothesized that PTPN3 might interact in vivo with the HBc CTD of HBV capsids through a PDZ/PBM interaction.

Indeed, several data indicate that at least a fraction of the HBc CTD is exposed on the external surface of the particle^57–59^. The CTD of cytosolic HBc is responsible for binding and encapsidating viral pgRNA through its CTD. It is generally accepted that the arginine-rich C-terminal region of the HBc CTD interacts with the viral genome inside the particle. Once the viral genome is matured to rcDNA within the capsids, a fraction of the rcDNA-filled capsids is transported back to the nucleus to maintain the levels of cccDNA. At this step, the NLSs of the CTD are required. Therefore, to perform these functions, the CTD should be able to shuttle between the interior and the exterior of the capsids. Previous observations support this model^60,61^. In addition, the linker at the boundary between the assembly and arginine-rich domains of HBc seems to be mobile and may allow a large mobility of the C-terminal domain^62^.

Overexpression of a protein may affect other proteins or pathways due to non-physiological expression level. Thus, PTPN3 knockdown experiments were performed in infected HepG2-NTCP cells. The results indicate that PTPN3 knockdown promotes HBV infection-mediated cell death (Supporting Information Fig. S2a). PTPN3 knockdown resulted in reduced cell growth rates as compared to the non-target shRNA control (Fig. S2b). Only under PTPN3 knockdown, a HBV load-dependent decrease of cell viability is observed (Fig. S2c). Furthermore, the quantification of HBV infected cells demonstrated dose-dependent infection rates of cells transduced with a non-targeting shRNA, and that shNTCP significantly reduced HBV infection rates (Fig. S2d). However, HBV infection rates of cells transduced with shRNAs targeting PTPN3 are reduced. This result suggests that PTPN3 might be involved in cell survival, which has been reported previously^7^.

**Figure 6:**
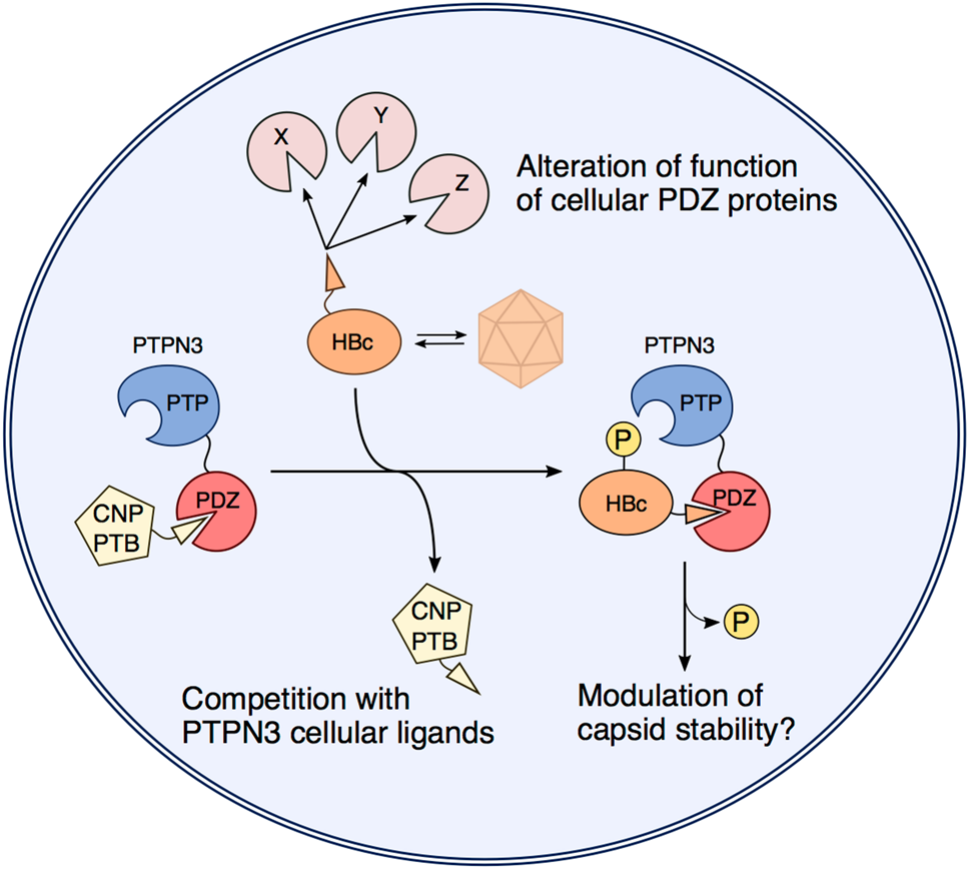
Schematic model of the multiple PBM-mediated interactions of HBc with PTPN3 and other PDZ-containing proteins. The model illustrates the competition of HBc with PTPN3 cellular ligands (CNP, PTB). The subsequent complex formed between the two proteins could allow the dephosphorylation of HBc by PTPN3, potentially affecting capsid stability. HBc could also target and alter functions of other cellular PDZ-containing proteins (proteins X, Y, Z).

Interestingly, PTPN3 contains 3 functional NLSs (1 in the FERM domain, 1 in the linker between the FERM and PDZ domains, and 1 in a loop of the PTP domain), and PTPN3 lacking its FERM domain is found in the nucleus^5^. Three isoforms of PTPN3 are produced by alternative splicing, two of which have truncated FERM domains. PTPN3 might play a role in the DNA import into the nucleus through the PBM-HBc/PTPN3-PDZ interaction and the NLSs. Another attractive possibility is a modulation of capsid stability involving the tyrosine dephosphorylation activity of PTPN3. The PBM-HBc/PTPN3-PDZ interaction would allow PTPN3 to dephosphorylate the tyrosine of HBc (Fig. 6). Several tyrosines in HBc are susceptible of being phosphorylated, including the tyrosine 132 (Y132), which is exposed in the HBc capsid and involved in capsid assembly^35^. To our best knowledge, there is currently no data reported in the literature on tyrosine phosphorylation in HBc. We can hypothesize that capsid assembly is regulated by phosphorylation of Y132, and that PTPN3 could dephosphorylate HBc and modulate the stability of the capsid (Fig. 6). The dephosphorylation of HBc by PTPN3 may lead to the destabilization of RC-DNA containing capsids before they reach the nucleus and thus interfere with cccDNA formation. Additionally, PTPN3 may be involved in HBV transcription via an HBc-dependent or -independent mechanism. HBc has been shown to be recruited on the cccDNA and is believed to impact HBV transcription. One might hypothesize that dephosphorylation of HBc can favor its binding to the cccDNA, thus increasing its transcription. Further studies are still needed to determine whether the increase of HBV RNA levels in PTPN3 overexpressing cells is transcriptional or post-transcriptional. Finally, we also observed an increase of HBV secretion that exceed the increase of HBV RNA level, suggesting an impact of PTPN3 on HBV secretion. HBc is involved in envelope protein recruitment and its phosphorylation status could affect this step. Altogether these results suggest that post-translational regulation of HBc may be required for the fine-tuning of HBc activities during the different steps of HBV replication.

In this study, we reported a pool of human PDZ-containing proteins potentially targeted by PBM-HBc (Fig. 6). Despite the non-canonical composition of the PBM sequence (i.e. a cysteine at the last C-terminal position), a large panel of interactors with high-to-medium affinities for PBM-HBc was detected in vitro. As expected, we identified GIPC1 and PTPN3, but also new cellular partners potentially involved in the HBV life cycle. Notably, a large majority of these binders are targeted by other viruses. For example, the three proteins DLG1, MAGI-1, and MPDZ are all targeted by the oncoviruses HBV, HPV 16 and 18, and HTLV-1, as well as by Adenovirus. Thus, viruses with quite distinct replication cycles encode PBM-containing proteins that target a common subset of cellular PDZ proteins during infection^52^. A majority of these PDZ-containing proteins are involved in cell–cell junction or polarity and cellular trafficking, suggesting that interfering with these functions is a strategy shared by viruses. The interaction of viral PBMs with PDZ-containing proteins frequently leads to a loss of function either by abnormal sequestration in cell structures or by proteasomal degradation, while in some cases, an apparent gain of function of the cellular protein is observed. In all cases, the targeting of PDZ-containing proteins and the alteration of cellular processes regulated by these proteins are likely to improve viral replication, dissemination in the host, or transmission to new hosts (Fig. 6).

As the structural data on PDZ domains in complex with a C-terminal cysteine PBM are rather sparse, we have solved the high-resolution structure of the PTPN3-PDZ/PBM-HBc complex. The atypical PBM-HBc binds PTPN3-PDZ establishing a network of interactions typical of class I PDZ domains despite the cysteine in the last C-terminal position, which explains the binding affinity for PTPN3-PDZ being comparable to the one of canonical PBMs. Interestingly, PTPN3 is also able to bind to the cytoplasmic domain of TACE (tumor necrosis factor alpha-convertase), which also presents a C-terminal cysteine in its PBM^21^, and GIPC1, the other reported PBM-HBc target, interacts as well with the human lutropin receptor, which also presents a C-terminal cysteine in its PBM (-YTEC_COOH_) sequence. Structural insights about the binding of a cysteine at the C-terminal part of a PBM to a PDZ domain have been reported for a class II PBM (liprin-α peptide) with the GRIP1 PDZ6^63^. We compared the binding mode of this complex with our crystal structure of the complex between the class I PBM of HBc and PTPN3-PDZ, and we found a similar orientation of the side-chains of the two cysteines in the hydrophobic pocket. The position of these side-chains is equivalent to the one of the classical C-terminal hydrophobic leucine in the PBM-16E6 bound to PTPN3-PDZ. Thus, it appears that a C-terminal cysteine in PBMs is not discriminant, since PDZ domains such as PTPN3-PDZ can interact with these PBMs and with canonical ones displaying a similar network of interaction and affinity.

HBV, through the PBM-HBc, may enter in competition with endogenous partners to hijack signalling pathways in infected cells as already reported for the rabies glycoprotein^30^. We propose a list of PBM-containing candidates that are possible partners of PTPN3 through PDZ/PBM interactions using a pull-down assay (Fig. 6). Among the candidates, CNP, for example, appears to be involved in cellular response against HBV infection, as it has been found to block viral protein synthesis and reduce viral RNAs. PTPN3 might participate in a signalling pathway of interferon-stimulated genes. HBc could block this process by targeting the PDZ domain of PTPN3 partner and displacing CNP. Similarly, HBc could displace PTB, another PBM partner of PTPN3. PTB binds the regulatory protein PRE, and both proteins appear to enhance the nuclear export of unspliced RNAs (such as the HBV RNAs). By targeting and disrupting the PTPN3-PTB interaction, HBV could potentially favour the export of its RNAs, and thus enhance virus production.

In conclusion, during infection, the virus may target endogenous proteins for a direct and specific action as PTPN3 on encapsidation, but also for a more general perturbation of cellular homeostasis in its favor (Fig. 6). Further research on the specific set of PDZ-containing proteins targeted by the PBM of HBc and the PBM-containing proteins interacting with PDZ-PTPN3 will provide insights into the mechanisms whereby the interaction of HBc with these cellular proteins is advantageous for the HBV life cycle. Our integrative study identifies perturbations at the levels of proteins and cellular pathways and provides a new resource for the research on HBV-host interactions.

## Materials and methods

### Production and purification of recombinant proteins and PBM peptides

PTPN3-PDZ is encoded as an N-terminal glutathione S-transferase (GST) tagged protein. Construct delimitations of PTPN3-PDZ are from residues 489 to 597 except for NMR titration with PBM-HBc peptide and Holdup assays where the construct originated from another plasmid and its delimitation was from residues 504 to 597. Uniformly ^15^N-labeled and unlabeled PTPN3-PDZ constructs were expressed and purified as previously described^25^. For pull-down assays with GST-PTPN3-PDZ, the cleavage step by TEV protease in the protocol was skipped. The samples for crystallogenesis of PTPN3-PDZ were prepared as previously described^25^.

The HBc expression vector pRSF-T7-HBc183opt plasmid previously described^64^ carrying the HBc gene, optimized for bacterial expression, was used to express the full-length HBc. Transformed *E. coli* BL21 CodonPlus (DE3) cells were grown in LB medium with 100 µg/mL of kanamycin and 25 µg/mL of chloramphenicol. Protein expression was induced at an optic density at 600 nm of 2 with 1 mM isopropyl thio-β-d-galactoside at 18 °C for 16 h. Harvested cells were resuspended in buffer B (50 mM Tris/HCl, pH 7.5, 300 mM NaCl, 5 mM DTT), 1 mg/mL lysozyme, 50 U/mL benzonase and protease inhibitor cocktail (ROCHE), and then incubated 45 min with 0.5% Triton X-100. The lysis is achieved by lysozyme and sonication. The cell lysate was cleared by centrifugation at 20,000*g*. HBV core particles were further purified by 10–60% sucrose gradient (prepared with buffer B) ultracentrifugation at 140,000*g* for 3 h (Optima L-80, BECKMAN ultracentrifuge). The eluted fractions containing the protein were pooled, and ammonium sulfate was then added to 40% saturation^65^. The mixture was left for 1 h to salt out the HBV core and then centrifuged at 20,000*g* for 15 min to pellet the HBV core. Minimal volume of buffer B was added to resuspend the pellet, and then the mixture was dialyzed against buffer B overnight. The purified HBV core sample was concentrated by ultra-filtration with a MW cut-off of 100 kD (MILLIPORE, USA). Protein concentration was estimated by the Bradford method.

The peptides PBM-HBc and biotinylated PBM-HBc were synthesized in solid phase using Fmoc strategy. They are purified (> 98% by HPLC) and quality controlled by mass spectrometry and HPLC (PROTEOGENIX) and resuspended in H_2_O.

### Crystallisation, data collection, and structure determination

The PBM-HBc peptide used for co-crystallization was added in excess to form > 95% of the complex with the protein. The PDZ domain-peptide complex for crystallization was generated as previously reported^25^. The best crystals were obtained by mixing 200 nL of PTPN3-PDZ·PBM-HBc complex solution (concentration of the PDZ domain at 4.5 mg/mL) in 20 mM HEPES pH 8, 150 mM NaCl, 0.5 mM Tris(2-carboxyethyl)phosphine (TCEP) mixed with 200 nL of reservoir solution containing 20% w/v PEG 3350, 0.2 M NaBr at pH 7. Crystals were then flash-cooled in liquid nitrogen using Paratone-paraffin 50%(v/v)/50%v/v) oil as the cryoprotectant.

X-ray diffraction data were collected at a wavelength of 0.979 Å on the beamline PROXIMA-1 at Synchrotron SOLEIL (St. Aubin, France). The data were processed, and the structures were solved as previously reported^25^ using the search atomic model of PTPN3-PDZ (PDB ID: 6HKS). The positions of the bound peptides were determined from a *F*_*o*_–*F*_*c*_ difference electron density maps. Models, refinement, and the overall assessment of model quality were done as previously described^25^. The crystal parameters, data collection statistics, and final refinement statistics are shown in Table 1. All structural figures were generated with the PyMOL Molecular Graphics System, Version 1.7 (Schrödinger).

### Analytic ultracentrifugation (AUC) experiments

HBc CLPs formation was verified by AUC. Sedimentation velocity experiments were carried out at 20 °C using an analytical ultracentrifuge (BECKMAN COULTER OPTIMA AUC) equipped with a AN50-Ti rotor. The HBc protein sample at approximately 150 mg/mL was centrifuged for 17 h at 12,000 rpm. Interference profiles were analyzed with SEDFIT 16.1^66^ using a continuous size distribution c(S) model with a constant diffusion coefficient D.

### NMR experiments

The NMR samples of the PTPN3-PDZ constructs were prepared in buffer A with 0.5 mM TCEP and D_2_O (5–10% v/v). The NMR titration experiments to map the PTPN3-PDZ·PBM interaction were performed on a 600-MHz VARIAN NMR System spectrometer equipped with a triple resonance ^1^H{^13^C/^15^N} cryoprobe at 15 °C as previously reported^25^ with a stock solution of the unlabeled peptide PBM-HBc of 3.6 mM at pH 7.5 and a sample of ^15^N-labeled PTPN3-PDZ initially containing 260 μL at a concentration of 95 μM. The chemical shift changes were followed with the CcpNmr Analysis software^67^.

The NMR binding experiments between PTPN3-PDZ and full-length HBc as CLPs were performed at 20 °C on a 600-MHz BRUKER Avance III HD spectrometer equipped with a cryoprobe. Briefly, the unlabeled HBc (stock solutions at 7.1 mM) were added in a sample initially containing 270 μL of ^15^N-labeled PTPN3-PDZ at a concentration of 95 μM. A series of ^1^H, ^15^N HSQC-TROSY spectra was recorded for different titration points with a ratio PDZ:HBc (mol:mol) 1:0, 1:1.4, 1:5.6, and 1:19.4.

### Cells

HeLaS3-HBc and HeLaS3-HBcY132A were maintained in Dulbecco’s modified Eagle’s medium (DMEM) with 10% fetal calf serum (FCS). They are derived from HeLaS3 and express the HBc wild type protein or HBc Y132A mutant, respectively, as a fusion with Flag and HA Tags. They have been established as previously described^68^.

HepG2-NTCP cells (A3 clone) derive from HepG2 cells and express the human sodium taurocholate cotransporting polypeptide (NTCP). HepG2-NTCPsec+ cells derive from HepG2 and express NTCP. They have been established to allow the amplification of infectious HBV^69^. HepG2-NTCP cells are grown in DMEM with 10% fetal calf serum (FCS). HepG2-NTCP-PTPN3 cells are derived from HepG2-NTCP cells, and stably express PTPN3. pCMV6-PTPN3 plasmid was from ORIGENE (reference RC15851).

### Pull-down assays

The construct PTPN3-PDZ tagged with a N-terminal GST (GST-PTPN3-PDZ) was expressed and purified as previously described^25^ without TEV cleavage. The additional TEV cleavage step of GST-PTPN3-PDZ provides the GST alone. Pull-down assays were performed as previously described^31^ except that the lysate is from human cervical carcinoma cell line HeLa S3 cells overexpressing the HF-HBc.

Briefly, HeLa S3 cells were prepared as followed. Cells overexpressing the HF-HBc protein were lysed in 20 mM Tris–HCl pH 7.5, 200 mM NaCl, 0.5% Nonidet P-40, complete protease inhibitor cocktail (ROCHE), 2 mM DTT by incubating cells on ice for 15 min followed by five sonication cycles and centrifugation at 13,000*g* at 4 °C for 20 min.

After lysate binding, the glutathione-agarose beads were washed four times with binding buffer, pelleted, and resuspended in SDS-PAGE sample buffer for Western blotting. After boiling, the bound proteins were analyzed using 12% SDS-PAGE followed by Western blotting using HRP-conjugated HA Epitope Tag monoclonal antibody (26183-HRP, THERMOFISHER) diluted to 1:2000. The proteins were visualized by enhanced chemiluminescence according to the manufacturer's instructions (Immobilon Forte Western HRP substrate, MILLIPORE).

For pull-down analyses by LC–MS/MS, the proteins bound to the glutathione-agarose resin were eluted by incubation with binding buffer containing 10 mM glutathione for 1 h at 4 °C. The beads were separated by centrifugation, and the proteins in the supernatant were precipitated using trichloroacetic acid/acetone^70^.

Samples were re-suspended in 300 µL 8 M urea/100 mM Tris HCl pH 8.5. Briefly, samples were reduced with 5 mM TCEP for 30 min at room temperature and alkylated with 10 mM iodoacetamide for 30 min at room temperature in the dark. Then, proteins were digested for 5 h at 30 °C with 500 ng rLys-C Mass Spec Grade (PROMEGA, Madison, WI, USA). Samples were then treated as previously described^71^ as well as tryptic peptides analysis^71^ with minor changes.

All data were searched using Andromeda^72^ with MaxQuant software^73,74^ version 1.5.3.8 against Homo sapiens reference proteome from Uniprot (20,399 entries) concatenated with GST-PTPN3-PDZ protein sequence, usual known mass spectrometry contaminants and reversed sequences of all entries as previously described^71^. The “match between runs” feature was applied between replicates with a maximal retention time window of 0.7 min. One unique peptide to the protein group was required for the protein identification. A false discovery rate (FDR) cut-off of 1% was applied at the peptide and protein levels. Reverse proteins and usual MS contaminants were removed before the analysis of the data. Quantification of each identified protein was performed by summing the intensities of its associated peptides.

### Native Western blot

Cells were lysed in 1X Native Sample Buffer (THERMOFISHER) supplemented with 1% N-Dodecyl β-D-maltoside. After centrifugation (45 min, 13,000*g*, 4 °C), samples were supplemented with 0.25% of G-250 (THERMOFISHER) and loaded in a Native PAGE 3–12% Bis–Tris gel (THERMOFISHER). Electrophoresis was run at 150 V using NativePAGE Running Buffer Kit (THERMOFISHER) following the manufacturer protocol. Samples were electro-transferred to nitrocellulose membranes using transfer solution (2X Tris–Glycine; 7.5% EtOH; 0.02% SDS). Blots were incubated with the indicated primary antibodies and then probed with HRP-conjugated secondary antibodies.

### HBV production and infection

For virus production, HepAD38 cells were grown in Williams E medium supplemented with 5% FCS, 7.10^–5^ M hydrocortisone hemisuccinate, 5 mg/mL insulin, and 2% dimethylsulfoxide (DMSO). HBV particles were concentrated through ultracentrifugation with a SW-32 rotor at 32,000 rpm and a 20% sucrose cushion. Titers of enveloped DNA-containing viral particles were determined by immunoprecipitation with an anti-preS1 antibody (gift of C. Sureau), followed by qPCR quantification of viral RC-DNA using RC primers RC 5′ (5′-CACTCTATGGAAGGCGGGTA-3′) and RC 3′ (5′-TGCTCCAGCTCCTACCTTGT-3′). Enveloped DNA-containing viral particles (vp) quantification was used to normalize for virus infection, and multiplicities of infection (MOI) were expressed as viral particles per cell.

HepG2-NTCP or HepG2-NTCP-PTPN3 cells were infected with normalized amounts of virus at a MOI of 100 in presence of 4% PEG 8000 and 3% DMSO. Infected cells were maintained in medium supplemented by 3% DMSO and collected 7 days after infection for virus replication analysis.

### Quantitative RT-PCR (RT-qPCR)

Total RNA was prepared using TRIzol reagent (INVITROGEN), and DNA contamination was removed by TURBO DNase treatment (AMBION). RNA (500 ng) was retrotranscribed using Oligo dT primers and RevertAid H Minus M-MuLV reverse transcriptase (FERMENTAS). RT-qPCR experiments were carried out as described^75^. Relative quantifications were performed as described previously^76^. The primers HBV RNAall-F and HBV RNAall-R amplify all HBV transcripts (pgRNA as well as the 2.4 and 2.1 kb mRNA) except the 0.8 kb transcript encoding HBx.

### Quantification of cccDNA

For nuclei isolation, cells were lysed in fractionation buffer containing 100 mM tris HCl pH 7 and 0.375% NP40 and shook using a vortex for 30 s. Nuclei were pelleted by centrifugation (10 min at 15,000 rpm) and washed in fractionation buffer. Supernatant corresponding to cytoplasmic fractions were kept for RC-DNA quantification. Following centrifugation, DNA was extracted using QIAamp DNA blood mini kit (QIAGEN). For HBV cccDNA quantification, DNA was pre-treated with 10 U of plasmid-safe DNase (EPICENTER) for 1 h at 37 °C, and cccDNA was amplified using cccDNA primers: cccDNA 5′ (5′-GTGCACTTCGCTTCACCTCT-3′) and cccDNA 3′ (5′-AGCTTGGAGGCTTGAACAGT-3′). Samples were normalized using CyclinA2 quantification by qPCR with the following primers: CCNA2 5′ (5′-CCTGCTCAGTTTCCTTTGGT-3′) and CCNA2 3′ (5′-AGACGCCCAGAGATGCAG-3′).

### Quantification of RC-DNA in the cytoplasm

Supernatant from nucleocytoplasmic fractionation described above is digested with DNase and RNase for 1 h at 37 °C in presence of 6.25 mM MgOAc to remove nucleic acid contaminants. Capsids were then digested with 7.5 µL Proteinase K (EUROBIO) and 12.5 mM EDTA, 1% SDS, 125 mM NaCl. DNA was then purified using phenol–chloroform technique followed by isopropanol precipitation and quantified by qPCR using RC primers: RC 5′ (5′-CACTCTATGGAAGGCGGGTA-3′) and RC 3′ (5′-TGCTCCAGCTCCTACCTTGT-3′).

### Virus secretion

Supernatant from infected cells were recovered and centrifuge 1 min at 13,000*g* to remove cellular debris, and PEG 8000 was added to a 5% final concentration. After precipitation at 4 °C, supernatants were centrifuged 15 min at 13,000*g*. Pellets containing virions were resuspended in lysis buffer containing 1% SDS, 0,1 M NaHCO_3_, 0,1 M TrisHCl pH 6.5 and 0.8 mg/ml proteinase K and incubated 1 h 56 °C. DNA was then purified using phenol–chloroform technique followed by isopropanol precipitation and quantified by qPCR using RC primers: RC 5′ (5′-CACTCTATGGAAGGCGGGTA-3′) and RC 3′ (5′-TGCTCCAGCTCCTACCTTGT-3′).

### Knockdown of PTPN3 by lentiviral transduction

Knockdown of PTPN3 by lentiviral transduction was performed as previously described^69^. Briefly, HepG2-NTCPsec+ cells were seeded in 384-well assay plates, transduced with lentiviral particle at MOI 10 of PTPN3-targeting sequences CCGGCGTGTGTATGAAGAAGGTTTACTCGAGTAAACCTTCTTCATACACACGTTTTTG (sh003), CCGGTGACACCACCCGGGTATTATTCTCGAGAATAATACCCGGGTGGT GTCATTTTTG (sh004), TRCN0000320833, TRCN0000320836 (MISSION, SIGMA-ALDRICH, St Louis, MO, USA), NTCP, or a non-targeting shRNA control. Transduced cells were selected with puromycin followed by inoculation with HBV. Cell growth was determined by microscopic counting of cell nuclei stained with Hoechst33342. HBV infection was analyzed by immunofluorescence staining and quantification of HBcAg expressing cells.

### Holdup assay

The holdup assay was carried out against the biotinylated peptide PBM-HBc (peptide sequence -RRRRSQSRESQC) in duplicates as previously described^48, 49^ with minor modifications. We measured PBM-HBc interactions against 255 human PDZ domains. The minimal binding intensity (BI) threshold value is 0.2 to define a significant interaction, as previously reported^48^.

## Supplementary Information

Supplementary Information.

Supplementary Table 1.

Supplementary Table 2.

## Data Availability

All data generated or analysed during this study are included in this published article.
