# Supplementary material for: Molecular basis of the interaction of the human tyrosine phosphatase PTPN3 with the hepatitis B virus core protein
Source: Sci Rep. 2021 Jan 13;11:944. doi: 10.1038/s41598-020-79580-9 (PMC7806630; doi:10.1038/s41598-020-79580-9)
Supplement: Supplementary file 1 — Supplementary Information. [file 41598_2020_79580_MOESM1_ESM.docx]

Supporting Information

**Molecular basis of the interaction of the human tyrosine phosphatase PTPN3 with the hepatitis B virus core protein**

Mariano Genera^1,2^, Barbara Quioc-Salomon^3,4,5^, Antonin Nourisson^1^, Baptiste Colcombet-Cazenave^1,2^, Ahmed Haouz^6^, Ariel Mechaly^6^, Mariette Matondo^7^, Magalie Duchateau^7^, Alexander König^8^, Marc P. Windisch^8^, Christine Neuveut^3,4^, Nicolas Wolff^1^ and Célia Caillet-Saguy^1,*^

^1^ Channel-Receptors Unit, Institut Pasteur, UMR 3571 CNRS, 75015, Paris, France; ^2^ Sorbonne Université, Complexité du Vivant, F-75005 Paris,  France; ^3^ CNRS, UMR 3569, 75015 Paris,  France; ^4^ Department of Virology, Institut Pasteur, Paris, France; ^5^ Université Paris Diderot, Sorbonne Paris Cité, Paris,  France; ^6^ Crystallography Platform-C2RT, Department of Structural Biology and Chemistry, CNRS UMR-3528, Institut Pasteur, 75015 Paris, France; ^7^ Proteomics Platform, Mass Spectrometry for Biology Utechs (MSBio), USR 2000 CNRS, Institut Pasteur, F-75724 Paris, France; ^8^ Applied Molecular Virology Laboratory, Institut Pasteur Korea, 696 Sampyung-dong, Bundang-gu, Seongnam-si, Gyeonggi-do, South Korea.

* Correspondence should be addressed: Célia Caillet-Saguy: Unité Récepteurs-Canaux, Institut Pasteur, F-75724 Paris, France; [celia.caillet-saguy@pasteur.fr](mailto:celia.caillet-saguy@pasteur.fr); Tel. +33(0)1 44 38 91 81; Fax. +33 (0)1 45 68 88 34.

Supporting Information Figure S1. Full-length Western blots of figure 3.

**Panel A**


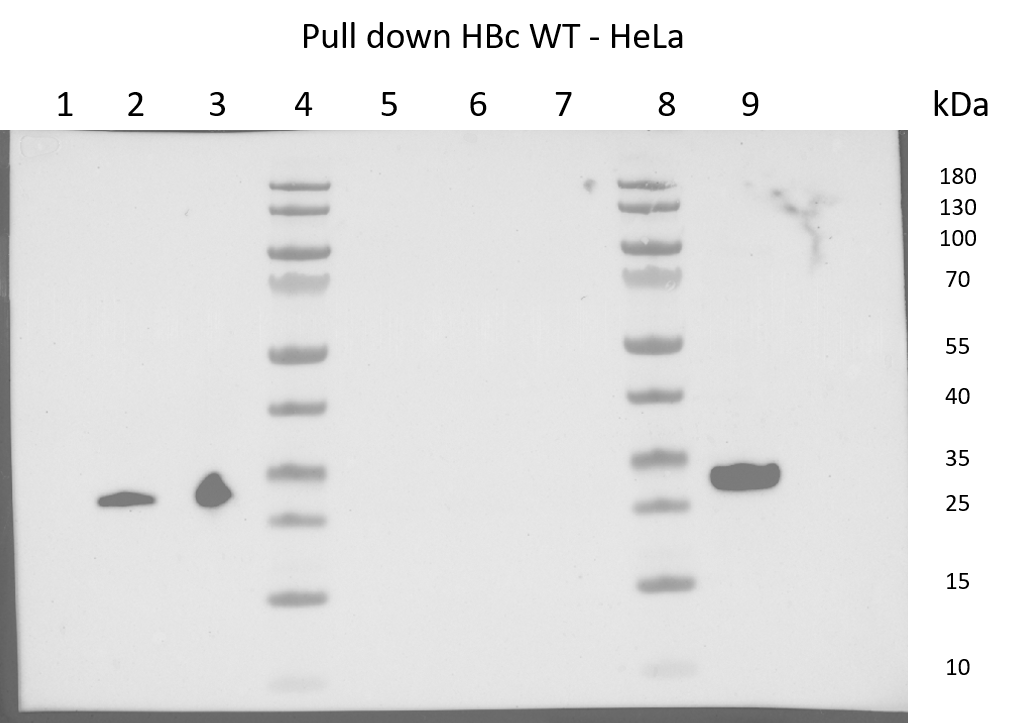


Figure caption: GST pull-down assay of GST-tagged PTPN3 PDZ domain against HeLa S3 cell lysate overexpressing wild type HBc. Lane 1: GST-PTPN3-PDZ in absence of lysate containing HA-HBc WT; Lanes 2 and 3: GST-PTPN3-PDZ in presence of 50 and 200 µg of total proteins from cell lysate containing HA-HBc WT respectively; Lane 5: GST in absence of lysate containing HA-HBc WT; Lanes 6 and 7: GST in presence of 50 and 200 µg of total proteins from cell lysate containing HA-HBc WT respectively; Lane 9 shows the signal of HA-HBc WT in 50µg of total proteins from HeLa S3 cell lysate; Lanes 4 and 8: molecular weight makers, the bands correspond to 180, 130, 100, 70, 55, 40, 35, 25, 15 and 10 kDa.


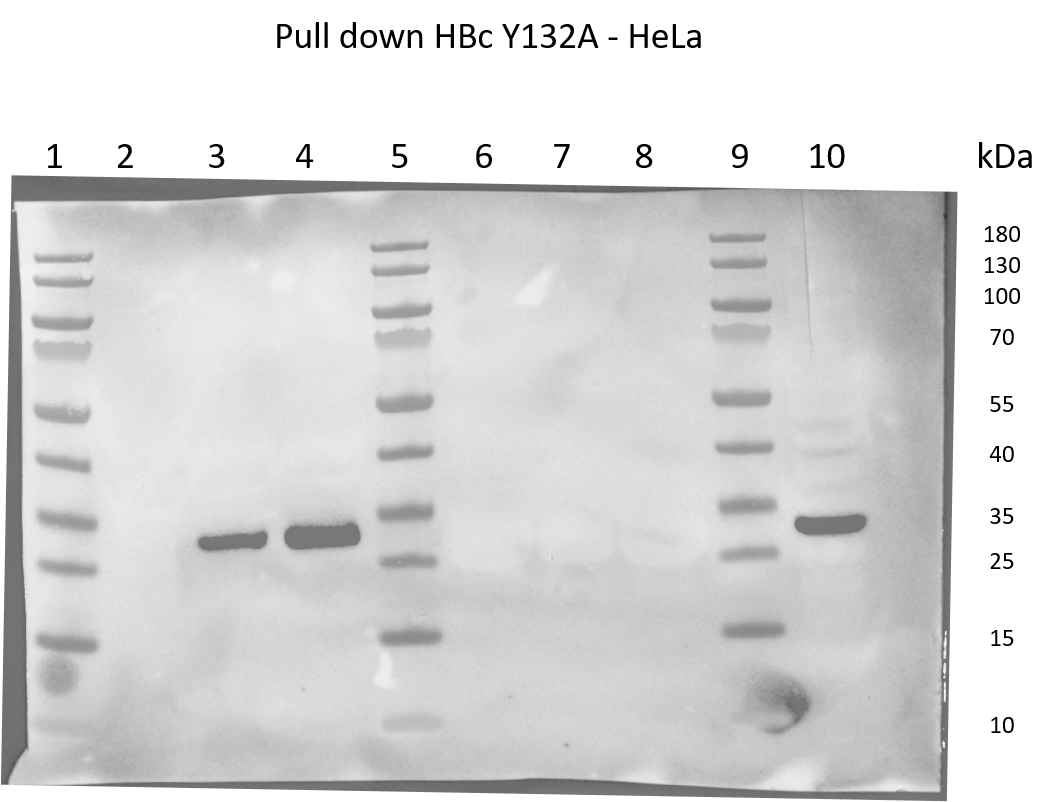


Figure caption: GST pull-down assay of GST-tagged PTPN3 PDZ domain against HeLa S3 cell lysate overexpressing HBc Y132A. Lane 2: GST-PTPN3-PDZ in absence of lysate containing HA-HBc Y132A; Lanes 3 and 4: GST-PTPN3-PDZ in presence of 50 and 200 µg of total proteins from cell lysate containing HA-HBc Y132A respectively; Lane 6: GST in absence of lysate containing HA-HBc Y132A; Lanes 7 and 8: GST in presence of 50 and 200 µg of total proteins from cell lysate containing HA-HBc Y132A respectively; Lane 10 shows the signal of HA-HBc Y132A in 50µg of total proteins from HeLa S3 cell lysate; Lanes 1, 5 and 9: molecular weight makers, the bands correspond to 180, 130, 100, 70, 55, 40, 35, 25, 15 and 10 kDa.

**Panel B**


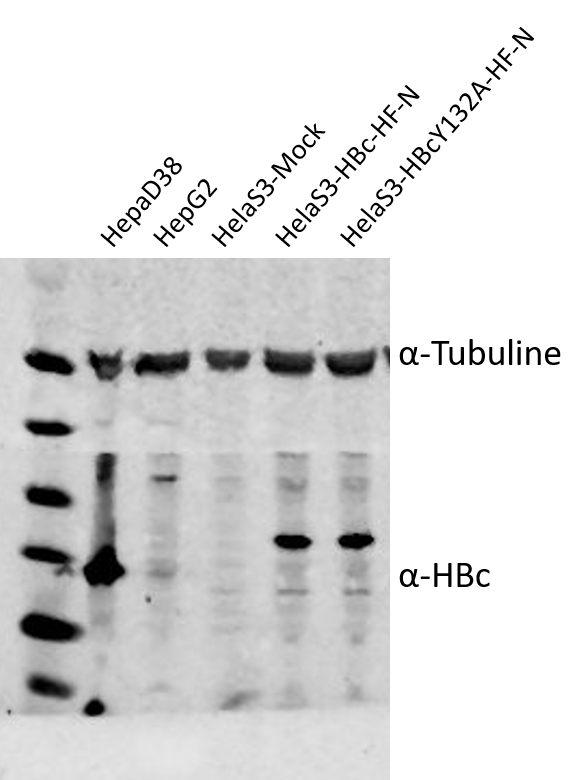


Figure caption: Western blot on the Hela S3 HA-HBc WT and HA-HBc Y132A using the anti-HA antibody (two last lanes). The HelaS3 cell lines express HBc with a HA-Flag tag which causes a migration higher


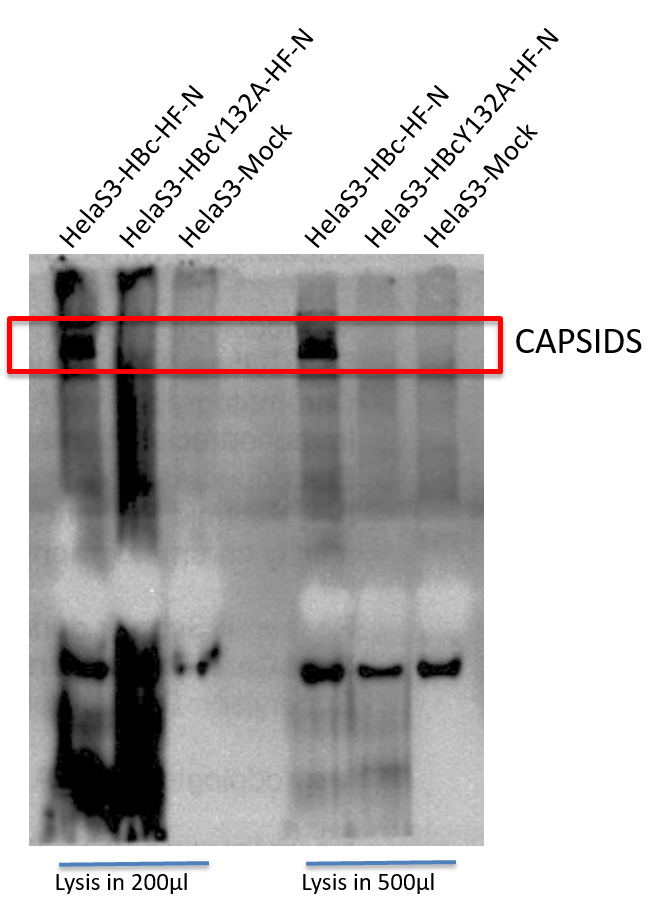


Figure caption: Native Western blot on the Hela S3 HA-HBc WT and HA-HBc Y132A using the anti-HA antibody.

Supporting Information Figure S2. PTPN3 knockdown promotes HBV infection-mediated cell death. (a) HepG2-NTCP cells were transduced with lentiviruses (shRNA) targeting three different PTPN3 sequences (sh003-005), NTCP, or a non-targeting shRNA control, followed by inoculation with serially diluted HBV (156-10,000 GEq/cell) or mock-infection. At day 7 post-infection, immunofluorescence staining was performed. HBc and cell nuclei are shown in green and blue, respectively. (b) Cell growth changes of non-HBV infected cells transduced with indicated shRNAs. (c) Cell viability normalized to the corresponding shRNA at mock-infection. (d) Quantification of HBV infection rates.


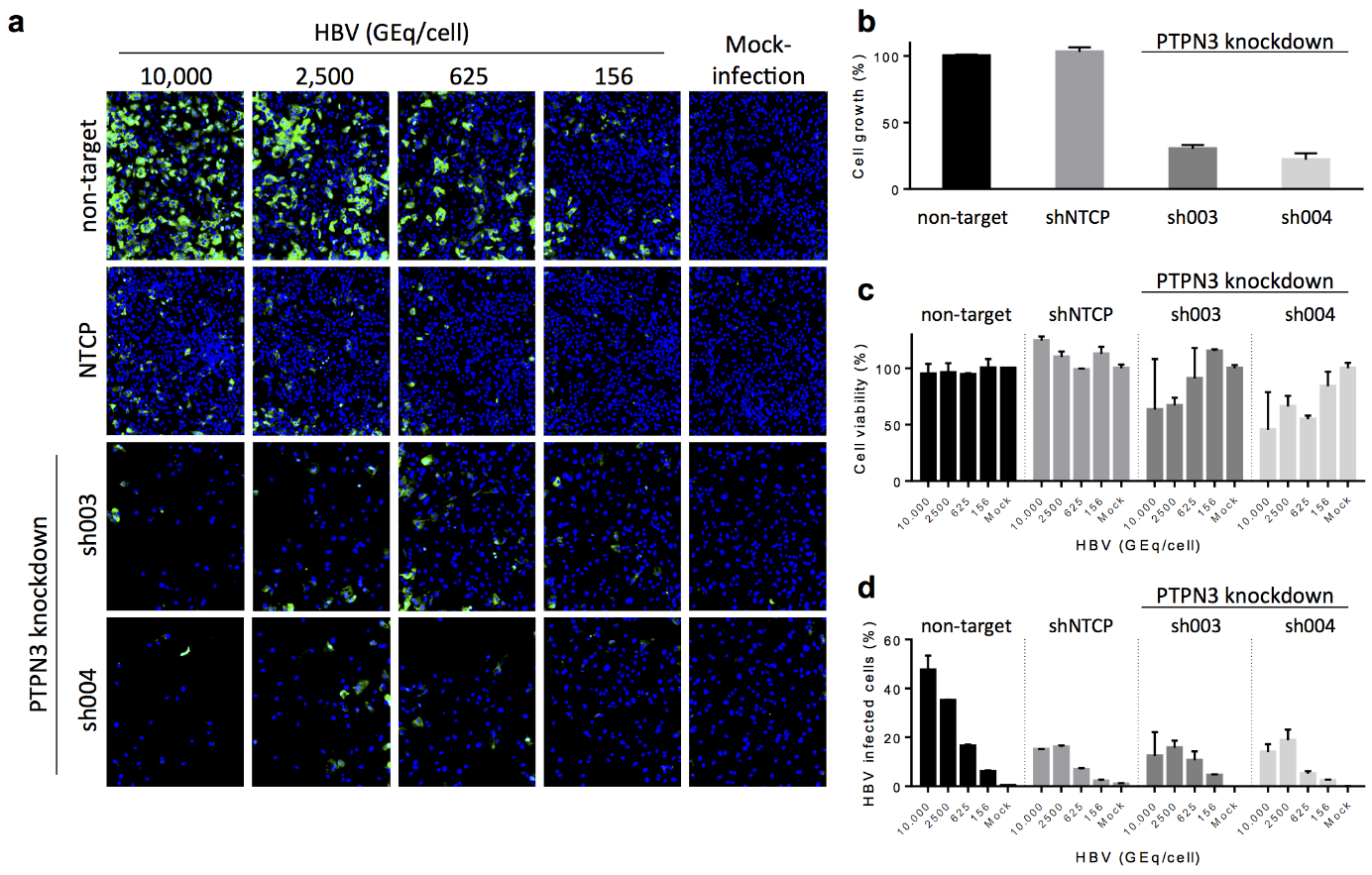


Supporting Information Table 1. Protein quantified by mass spectrometry of potential PTPN3-PDZ interaction partners. Listed proteins were exclusively detected in the sample GST-PTPN3-PDZ samples in the triplicate (absent in the GST controls).

See the excel file TableS1mass-spec.xlsx

Supporting Information Table 2. PBM-containing proteins detected by mass spectrometry of potential PTPN3-PDZ interaction partners. Listed proteins were ranked according to the PBM type from I to III.

See the excel file suptable2-pulldown-mass-spec-PTPN3-PDZ-PBMpartners-3classes
